# On the Pharmacology of Iron

**Published:** 1891-04-25

**Authors:** 


### THERAPEUTICS.

### ON THE PHARMACOLOGY OF IRON.

The very general use of the preparations of iron have led not only the practitioner, but his patient also, to recognise certain conditions as calling for its exhibition, and about its utility neither entertain a doubt. Some minor pharmaceutical considerations guide us in the selection of the particular preparation that we employ in individual cases, but in cases of anæmia and chlorosis the fundamental idea is that the blood corpuscles are deficient in hæmoglobin, and that the iron we administer is partly absorbed, and forms the source of the iron which goes to form the increased hæmoglobin.

It is, however, very doubtful if the inorganic iron salts as prescribed are ever utilised in the formation of hæmoglobin, except in a very indirect manner. Hæmoglobin contains about ·3 per cent. of iron, and the whole amount of iron in the blood of a man of average size and weight only amounts to about thirty-five to forty grains. Professor Tumas, of Warsaw, has recently reported the results of some experiments conducted with a view to ascertaining the action of iron in the formation of hæmoglobin when administered artificially. In some of his experiments, iron was administered to healthy dogs, in others an anæmic condition was induced by the abstraction of blood, varying in amount from about fifty-five to fifteen per cent. of the total quantity in the animals' bodies. From these experiments the Professor came to the following conclusions: (1) In the normal animals no increase in the number of red corpuscles nor in the percentage of hæmoglobin followed the introduction of iron, whether administered by the stomach or introduced subcutaneously; (2) in the cases of artificially induced anæmia, where the amount of blood abstracted did not exceed twenty-five per cent. of the total amount in the body, the administration of iron did not accelerate the regeneration of blood; but (3) when the amount of blood abstracted exceeded twenty-six per cent., the administration of iron greatly accelerated the return to the normal condition; (4) the hæmoglobin is restored far more rapidly than the regeneration of the red blood corpuscles; (5) the experiments appear to corroborate the generally accepted views that the administration of iron salts directly influence the reformation of blood by entering into the synthesis of hæmoglobin. These conclusions are corroborated by similar experiments conducted by Dr. Skvortzoff, who further ascertained that iron salts administered to healthy animals, while not increasing the amount of hæmoglobin, or the number of red corpuscles, actually increases the decomposition of fats and carbo-hydrates, and also diminishes the absorption of proteid food to some extent. Another interesting point observed was that in the cases in which iron was administered to dogs made anæmic artificially, the amount of iron absorbed varied with the needs of the economy, and was not in direct proportion to the amount administered.

On the other hand, the experiments of E. W. Hamburger, which were conducted with great care, would appear to prove that the inorganic salts of iron are not absorbed to any extent. A dog was fed on 300 grammes of meat a day containing 15 milligrammes of iron. At the end of twelve days, therefore, it had taken 180 milligrammes of iron in its food, and of this amount 176·5 milligrammes, that is, all but 3·5 milligrammes, reappeared in the excreta. Again, during the following nine days, 49 milligrammes of iron were added to the above-mentioned diet in the form of sulphate, and the original diet was then resumed for four more days. Thus, altogether 636 milligrammes of iron were taken in the course of the thirteen days, and of this 608·4 milligrammes were excreted. It was observed that the increased amount of iron excreted in the later portion of the experiment did not begin for some days after the addition of sulphates of iron to the food; this seems to indicate that iron, like manganese, is only absorbed, or at any rate more readily absorbed, when the cells of the mucous membrane have been acted on by the salt. It is recognised that metals are much more readily absorbed by the gastro-intestinal mucous membrane when it is in a catarrhal condition than when it is in a healthy state. Perhaps this fact may be held to explain the necessity for administering iron in doses far larger than are required to form the hæmoglobin.

It is, however, probable that Bunge has more correctly explained the beneficial action of inorganic iron salts in chlorosis, &c., without being absorbed. He reminds us that in chlorosis the abnormal state of the alimentary tract is generally a prominent feature, and that the gastric juice is poor in quality. Of the important function of healthy gastric juice as an antiseptic there can be no doubt, and consequently, when this physiological antiseptic becomes too weak to be efficient, various fermentation micro-organisms set up pathological changes in the food leading to the butyric fermentation, in which hydrogen is liberated, and in its nascent state reduces the compounds of sulphur in the food with the formation of alkaline sulphides. These alkaline sulphides decompose the organic compounds of iron in the food, forming sulphide of iron. In this manner the only absorbable form of iron, viz., the organic food compounds, are rendered useless as sulphides. The administration of inorganic salts of iron, by entering into combination with these alkaline sulphides, prevent the decomposition of the organic iron compounds, and their consequent loss to the economy. It is necessary that all the sulphides should be fixed in this manner, and the dose of iron must therefore be considerable.

Dr. Charles Taylor has recently reported in the *British Medical Journal* the results of his observations on a case of anæmia, undertaken with a view to seeing how much iron an anæmic person could take, and also whether the rapidity of progress would be hastened by the continuous administration of an iron salt. His patient was a very extreme case of anæmia, in a girl age 19, who had been getting gradually worse for two years. Having improved her digestive organs a little, he began to administer the iron, placing by her side a quart bottle of a solution of the tinct. ferri perchlor. with some spir. chloroformi and a tumbler, telling her to sip at it as much as she could day and night. During the first twenty-four hours as much as three pints were taken in this way. The strength was gradually increased from five minims per ounce to twenty-five minims, and she continued to get through about a quart a day. In the course of twenty-seven days no less than thirty ounces of the Brit. Pharmacopœia tinct. ferri perchlor. was consumed, and this without upsetting the stomach or necessitating the use of any stronger purgative than a pill of aloes and nux vomica daily. She improved most rapidly, and before she left the hospital, which she did in four weeks, was able to busy herself in the ward for the whole day without fatigue. Dr. Taylor rightly insists that the administration of small doses of iron in anæmia and chlorosis are less efficacious than the large, and his continuous method of giving large amounts is in accordance with the conclusions that will be drawn from the observations and experiments noted in the earlier portion of our article.

